# Background for Real-Time Monitoring and Intervention Related to Alcohol Use

**DOI:** 10.35946/arcr.v36.1.02

**Published:** 2014

**Authors:** Ellen Beckjord, Saul Shiffman

**Affiliations:** Ellen Beckjord, Ph.D., M.P.H., is an assistant professor in the Department of Psychiatry, and Saul Shiffman, Ph.D., is a professor in the Department of Psychology at the University of Pittsburgh Cancer Institute, Pittsburgh, Pennsylvania.

**Keywords:** Alcohol use, abuse, and dependence, alcohol use disorders, drinking behavior, risk factors, intervention, real-time assessment, ecological momentary assessment (EMA), real-time intervention, ecological momentary intervention (EMI), mobile devices, telecommunication, technology, electronic health technology

## Abstract

Real-time assessment, known as ecological momentary assessment (EMA), and real-time intervention (ecological momentary intervention [EMI]) can significantly extend the reach and impact of interventions to help individuals reduce their drinking behavior. For EMA, the user provides information on the variable of interest (e.g., drinking or craving) via a mobile device. This data reporting can occur either at pre-specified times or in certain high-risk situations. The primary benefits of EMA include external validity, minimized recall bias, and the ability to capture dynamic patterns in human behavior. EMI refers to interventions that are delivered via mobile devices at the time when the user needs it (i.e., in a high-risk situation). Key constructs of EMI are what interventions are delivered and when they are delivered. The timing of the EMI often is determined by the user’s EMA reports. Both EMA and EMI have been studied in people with alcohol use disorders. EMA and EMI often are used in conjunction with each other because EMA can help inform the optimal timing of EMI and help tailor its content. Further development of high-impact, algorithm-driven, technology-mediated real-time intervention may help reduce drinking and promote positive health behavior change.

Substance use disorders are challenging conditions to treat, and health care providers working with addicted individuals in an outpatient setting are at a disadvantage. They typically can spend perhaps 1 hour once a week with a patient, during which they can attempt to affect the patient’s substance use during the remainder of the week. Furthermore, in the case of alcohol use disorders (AUDs) (as defined by the American Psychiatric Association [2013]), providers usually have to rely on the patients’ self-reports of their drinking, their insights into the triggers that lead to it, and their assessment of its negative consequences. During the brief clinical encounter, the providers can relay educational information about the dangers of excessive drinking, use motivational interviewing techniques to encourage patients to attempt to reduce their drinking, and engage in problem solving with the patients to come up with strategies to avoid high-risk situations and reduce alcohol use. When the patients leave the clinical encounter, however, the health care providers can only hope that the patients will commit their discussions to memory and call upon them when they experience the urge to drink in the real world and that the conversations in the artificial safe haven of the consulting room somehow will have affected the patients enough to change their reactions in real-life, high-risk situations that challenge their commitment to change. In light of this, it is not surprising that the chances of achieving sustained abstinence are low and historically have been found to be as low as 15 percent or even less (Brandon et al. 2007; Helzer et al. 1985; Miller 1996).

Two factors contributing to these challenges facing providers trying to help individuals with drug addiction or alcoholism are the paucity of good information on a patient’s actual substance use behavior and an inability to intervene at the critical moments when the patient is making decisions about whether to drink or use other drugs. An enormous disconnect exists between the point of care, when the provider conducts an assessment and offers intervention during the clinical encounter, and the “point of choice,” when the patient makes the decision to drink or not to drink in real time. Methods of real-time data capture, such as ecological momentary assessment (EMA) (Shiffman et al. 2008), and real-time intervention, such as ecological momentary intervention (EMI) (Heron and Smyth 2010), offer ways to reduce this disconnect. However, the use of EMA and EMI does not fundamentally alter the conceptual or theoretical framework that may underlie the researcher’s or health care provider’s approach to understanding or treating substance use disorders. Rather, the use of these real-time approaches draws attention to the fact that the factors central to theoretical models of substance use and relapse prevention—including endogenous factors, such as craving and withdrawal, or exogenous factors, such as environmental cues (Marlatt 1980; Witkiewitz and Marlatt 2004)— are dynamic and vary over time. Accordingly, there is increasing support for approaches in research and treatment that measure and intervene in response to these factors in real time and in dynamic ways (Riley et al. 2011), especially because the feasibility of EMA and EMI approaches is increasing. Thus, today most people own an Internet-connected mobile device and keep that device on or near them nearly all the time (International Data Corporation 2013). As Collins and colleagues (1998) predicted in one of the earliest investigations using EMA to study alcohol use, “we anticipate that as electronic devices . . . become more common, data collection using hand-held computers will be more easily accommodated into the lives of research participants” (p. 314).

This article offers a rationale for why EMA is a powerful tool for behavioral scientists and explores some of the challenges and caveats associated with this approach. It also describes why EMI has strong potential to significantly improve the success rates of behavior change interventions. This discussion includes several examples of EMA and EMI in the context of alcohol use. Finally, the article provides guidance on how EMA and EMI can (and should) be used together as the foundation for a future of innovative, high-impact interventions to improve public health.

## EMA—An Overview

EMA is a family of methods to elicit data reporting in real time and in the patient’s real-life settings, usually via a mobile device (Shiffman et al. 2008; Stone and Shiffman 1994, 2002). The data most often are captured through screen-touch or text responses to prompts (Shiffman et al. 2008), but interactive voice responses also have been used to collect EMA data (e.g., Holt et al. 2012). EMA data collection procedures are defined by a protocol that usually includes prompts to the participant for responses to questions in real time (i.e., time-based sampling) and/or instructions to the participant to report specific events of interest (e.g., drinking episodes) in real time (i.e., event-based sampling) (Shiffman 2009; Shiffman et al. 2008). These two approaches often are used together. With both time-and event-based reporting, EMA protocols may ask directly about the behavior of interest with a forced-choice response option (“Are you drinking alcohol?” [yes/no]) or may offer a continuous response option (“How strong is your urge to drink alcohol?”) with responses possible on a 0 to 10 scale. Generally, and in studies of substance use specifically, compliance with EMA protocols is high, particularly for time-based sampling (Shiffman 2009). Compliance is harder to confirm for event-based sampling, but participant reports generally have been at a volume consistent with what would be expected given their baseline reports of substance use (Shiffman 2009). For example, in a study of adherence to EMA reporting among people addicted to tobacco, alcohol, cannabis, or opiates (Serre et al. 2012), compliance was substantial across all groups. Among those participants with AUDs, compliance with EMA data capture that prompted reports from participants four times per day averaged over 80 percent.

EMA protocols may focus exclusively on simply tracking a specific event of interest, such as drinking alcohol, but more often also collect contextual data, either through self-report or through data that can be passively sensed either by the mobile device itself (e.g., the participant’s location can be followed via GPS) or by another sensing device that may be connected to the mobile device (e.g., an ambulatory monitor that tracks the participant’s blood pressure). Ultimately, data collected via EMA are delivered to the researcher and/or health care provider. This may happen in real time by sending the data immediately from the mobile device to a server, or the data may be stored on the device until they are delivered on some regular basis.

### Strengths of EMA

EMA is a powerful method for behavioral research, and in particular the study of alcohol use, for several reasons. First, EMA has external validity because it assesses patients’ behavior in their natural environment. Compared with data collection in highly controlled laboratory settings or via study questionnaires completed by the participant at home, EMA captures data in real time and in highly localized contexts, thereby providing a picture of the participants’ experiences that more faithfully and comprehensively reflects their daily lives (Ferguson and Shiffman 2011; Reis 2010). Furthermore, the real-time sampling of participant experiences over time enables the researcher to make more valid inferences about the nature of a time-varying, episodic behavior of interest (e.g., drinking alcohol) as well as about the ways in which that behavior is associated with other behaviors (e.g., smoking) or contextual factors (e.g., mood, location) that are assessed via EMA as well. In this way, EMA offers a method for capturing repeated “snapshots” of the participants in their natural environments over time. When pieced together, these snapshots represent an accurate and rich observation of the participants’ daily lives, including how events and experiences flow over time and how they relate to a behavior of interest, such as drinking.

The second strength of EMA is that it avoids recall bias. Because EMA data are collected in real time, they are not subject to recall bias in the way that retrospective questionnaire data are (Bradburn et al. 1987). For many health behaviors, including drinking, there are known challenges associated with asking participants to accurately report how often they engaged in the behavior over even relatively short windows of retrospective recall. Even more challenging is the accurate reporting of contextual data associated with the target behavior of interest (e.g., what their mood was like the last time they drank alcohol). However, accurate reporting of contextual data can be critical to understanding the key antecedents of the target behavior of interest (Shiffman et al. 1996). The threats of recall bias to the validity of participant data are even greater when the behavior of interest, such as drinking alcohol, is likely to affect a participant’s cognitive abilities (Shiffman 2009). Particularly for time-varying behaviors and their time-varying contextual correlates, recall bias can significantly undermine the accuracy of inferences about the patterns of the behavior, its antecedents, and its consequences (Stone et al. 2007). EMA offers great benefits in this regard because the participants report on experiences as they occur and do not need to recreate associations that are better derived from the data.

The third, and possibly most important, strength of EMA is its ability to capture dynamic patterns in human behavior (Shiffman 2014; Smyth 2003; Stone et al. 2007). This strength is a function of the data yield, both in terms of how frequently EMA captures data on behaviors and outcomes of interest and in terms of the degree to which the data capture those behaviors and outcomes over a period of time. For example, consider a researcher using an EMA protocol that randomly prompts participants four times per day to report if they are drinking alcohol and that also includes an end-of-day report in which the participant reports the number of drinks he or she had that day. At each report of drinking, EMA may then be used to collect data on mood, the presence of others who also are drinking, self-efficacy, and the participant’s location (via GPS coordinates). At the end of the data collection period, the resulting set of EMA data will reveal numerous types of relevant information, including the following:

- How often drinking occurred;
- At what times drinking was most likely to occur;
- Which contextual factors were antecedents to drinking (e.g., if negative mood often preceded drinking);
- Modifiers of drinking behavior (e.g., whether the presence of others who were drinking affected the amount a person reported consuming during a drinking episode); or
- Consequences of drinking (e.g., whether self-efficacy was rated lower in reports that followed drinking episodes).

Importantly, EMA data also may reveal what processes may have led up to a drinking episode (e.g., whether emotional distress had been building up for hours or days before the episode) and what consequences followed the drinking episode (e.g., whether the individual experienced a hangover that put a damper on further drinking, or whether the person felt guilty). Thus, EMA data can help researchers identify, for example, different patterns of use as well as differences in the patterns in the target behavior between groups of participants (based on non–time-varying person-level covariates of interest, such as age or gender) (Stone and Shiffman 1994).

### Examples From the Alcohol Literature

Several studies have demonstrated the utility of EMA in deepening the understanding of alcohol use, its antecedents, and its consequences. Collins and colleagues (1998) used time-and event-based EMA reporting for 8 weeks to monitor individuals participating in an intervention to support drinking moderation, focusing on the role of affect in moderating drinking. As in many studies of alcohol use, the EMA protocol involved the reporting of a “drinking episode”—that is, the participants initiated the report when they began drinking and then could “close” the report when they were finished drinking, including a report of the number of drinks they had consumed during the episode. The investigators determined that positive mood before a drinking episode was associated with excessive drinking, whereas positive mood after a drinking episode was associated with fewer drinks during the episode (Collins et al. 1998). Given the role of affect in alcohol use and the time-varying nature of mood, several other studies have used EMA to monitor both drinking and mood to understand the relationship between the two. For example, following social drinkers for 2 weeks using EMA, Muraven and colleagues (2005) concluded that internal attributions for drinking more than intended were associated with worse mood reports, especially for women. And whereas Collins and colleagues (1998) found that positive mood after a drinking episode was associated with fewer drinks during the episode, Muraven’s study showed that negative mood after a drinking episode predicted more drinking during the next drinking episode, particularly for heavy drinkers. These studies demonstrate the utility of EMA in understanding the intricate relationships of antecedents and consequences with drinking behavior.

Other investigators have used EMA to characterize the timing of the relationship between negative affect and drinking. In their EMA study of 97 adults, Todd and colleagues (2009) found that negative mood early in the day was associated with shorter times to initiating drinking, but only among those who had high “drinking-to-cope” motivation (as determined via an assessment completed at baseline). Finally, in a more nuanced EMA study of affect and alcohol that followed underage drinkers for 3 weeks, Kashdan and colleagues (2010) demonstrated that among these underage drinkers, those who were better able to articulate their feelings during episodes of negative affect showed a weaker relationship between experiences of negative affect and subsequent drinking. The investigators hypothesized that drinking is more likely to serve as a coping mechanism for people who struggle with emotional differentiation.

EMA also has been used to examine the impact of other time-varying factors on alcohol use, such as environmental cues. In a study involving both time-based and event-based EMA protocols, Ramirez and Miranda (2014) showed that among adolescents who used alcohol, exposure to alcohol-related cues was associated with cravings, and such cue-provoked cravings were significantly higher among adolescents with more severe drinking problems.

Researchers also have used EMA approaches to gauge the impact of different interventions on drinking behaviors. Voogt and colleagues (2013), in a randomized controlled trial of college students who were heavy drinkers, followed participants relatively infrequently (i.e., 1 day per week) for more than 6 months during and after an intervention. By designing an EMA protocol that imposed a relatively low burden on participants, the researchers were able to capture more data over a longer period of time after the intervention portion of the trial ended. The protocol also distinguished between three different kinds of event-based outcomes, including overall alcohol consumption, binge drinking, and heavy drinking. The analyses concluded that the intervention was differentially effective over time in relationship to these outcomes. Thus, the effects of the intervention on frequency of binge drinking and heavy-drinking status lasted longer than its effects on weekly alcohol consumption (Voogt et al. 2013). Another EMA-based study analyzed the effectiveness of pharmacological treatment of alcohol dependence. In that study, Gurvich and colleagues (2013) tracked not only drinking episodes but also adherence to prescribed medications aimed at reducing drinking via EMA. It is important to note, however, that in such studies the EMA approach itself—that is, the repeated monitoring of drinking behavior—may act as an intervention that reduces drinking. This was demonstrated in a study by Ball and colleagues (2007), who conducted a three-group trial in which all three groups were exposed to EMA to monitor drinking. Two of the groups also received an actual intervention, whereas the third group was only exposed to EMA and served as a control. Although the investigators detected differences between the groups, all three groups reported significant decreases in their drinking over the course of the study, indicating reactivity to the EMA procedure itself and suggesting the presence of a “floor effect.” In another study of college-age drinkers, however, reactivity to EMA was minimal (Hufford et al. 2002). Thus, although researchers should consider the effects of EMA itself on drinking behavior in designing a study, the level of reactivity may vary by population.

EMA studies also have investigated how concurrent use of other substances, such as smoking, affects people’s subjective experiences of drinking. In a sample of 255 currently smoking frequent drinkers, Piasecki and colleagues (2012) found that participants rated alcohol as more pleasurable if they were smoking at the same time; furthermore, this relationship was stronger in women than in men. In another study of concurrent smoking and drinking for people in treatment for both, Holt and colleagues (2012) collected EMA data via interactive voice responses five times per day. In this population, the urge to smoke predicted smoking relapse, whereas the urge to drink did not predict drinking. The investigators hypothesized that the lack of an association between the urge to drink and drinking behavior may indicate that urges to drink arise more acutely in relationship to when drinking occurs, and that this close association may have been missed by their time-based EMA protocol. This conclusion could have implications for the design of EMA protocols in the context of alcohol use. Thus, to study such associations, it may be better to either use event-based EMA protocols that can proactively capture urges to drink or to conduct more frequent sampling within a time-based protocol.

To summarize, the use of EMA in alcohol research and treatment is feasible and has numerous potential applications. (For more information on EMA and its use in alcohol research, also see the article in this issue by Wray and colleagues.) It offers three primary benefits. First, it provides more externally valid data on drinking behavior, its antecedents, and its consequences by capturing data in real time, in individuals’ real-world environments. Second, it yields more valid data because it avoids recall bias. Third, high-frequency, well-distributed data capture allows for the derivation of complex and dynamic patterns and relationships between drinking behavior, endogenous factors, and environmental triggers. These benefits make EMA data an excellent foundation for determining what interventions are needed, and when—which are the core components of EMI.

## EMI

Whereas EMA uses mobile devices to collect information from patients, EMI uses such devices to deliver interventions to them. With this approach, patients can receive an intervention at any time, outside the clinical encounter, especially when they most need it—for example, when facing a high-risk situation and struggling to avoid violating a behavior-change goal.

The two key constructs important in the context of EMI are what interventions are used and when they are delivered (Heron and Smyth 2010). In general, it is less complicated to survey existing EMI-based interventions and decide which of them are likely to be most effective than it is to determine the best time for delivering them. The timing of real-time interventions can be based on schedules of varying complexity and ultimately may be most effective when it is informed by accompanying EMA-based data (Patrick et al. 2008). Without EMA, EMI schedules are limited to delivering interventions either at random times (in the hope that at least some of these intervention will occur close to a point of choice or that the interventions will have a more general nonlocal impact) or in response to user requests for help. Letting user demand exclusively drive real-time intervention delivery is problematic, however, because high-risk situations usually coincide with states of “hot cognition” when people are in states of severe craving and may be unlikely to ask for help (Loewenstein 2005).

The types of interventions delivered in real time via mobile devices are driven by the multimedia capabilities of most mobile devices that allow them to offer a variety of intervention content in multiple formats (Free et al. 2013; Kumar et al. 2013; Whittaker et al. 2009). Today, EMA and EMI studies most often use Internet-connected smartphones with embedded apps as a platform to deliver interventions; as a result, the potential intervention options are much greater than was possible in earlier investigations using less sophisticated devices. Ideally, any EMI interventions should adhere to the content of established evidence-based treatments. This requires translating components of existing evidence-based treatment (e.g., discussions about finding alternatives to alcohol for a person who is trying to reduce or stop drinking) into an intervention that can be delivered in real time on a mobile device (e.g., a text message encouraging the user to look for alternatives to alcohol). Unfortunately, most studies suggest that commercially available EMI programs, such as apps found in the iPhone or Android marketplaces, do not use evidence-based strategies (Abroms et al. 2011; Pagoto et al. 2013). To develop EMI programs that can reach the full potential of what real-time interventions have to offer, it is critical that developers focus on approaches that incorporate what is known about the optimal intervention for the target behavior in more traditional methods of delivery (Riley et al. 2011).

In their excellent review of EMI, Heron and Smyth (2010) have provided a comprehensive overview of the kinds of interventions offered via EMI in the context of health behavior change, even though some of the information may already be outdated now, given the pace at which EMI use is progressing in behavioral medicine. In general, EMI can be delivered in any format supported by the mobile device, including voice (e.g., connecting by phone to a person or a system that can deliver the intervention in real time or near–real time); images (still or video); messages; or longer types of educational or informational content (e.g., presentation of a PDF file containing specific information). The content of these interventions can vary widely. Heron and Smyth (2010) were able to categorize most EMIs as general behavioral treatment, cognitive– behavioral therapy, general cognitive treatment, feedback, motivational therapy, or psychoeducation. In many cases, EMI programs combine different intervention formats and content. Such combinations can be advantageous because they decrease the likelihood that the user will habituate to the EMI program, which might decrease its potential effectiveness.

Another way in which EMI content can vary is the degree to which the interventions are tailored to the individual user. Even outside the context of real-time interventions, evidence suggests that tailoring interventions can increase their effectiveness (Hawkins et al. 2008; Noar et al. 2007; Strecher et al. 2005). Tailoring can occur along multiple dimensions, including person-level factors such as age, gender, or race/ethnicity; self-reported level of motivation to change; the user’s most powerful triggers of the target behavior; or the user’s preferred coping strategies. Tailoring of the EMI content can also be a one-time event, determined by the user’s baseline characteristics before the start of the EMI program, or can be dynamic and be modified as the user’s needs or preferences change over time. Capturing the changing needs and preferences of the user may be accomplished by periodic measurements done via questionnaires, study visits, or continuous monitoring using EMA.

Most people seem to be receptive to EMI; in a review of existing studies, compliance with using a mobile device to receive EMIs usually was high, and participants generally rated EMI as credible and satisfying (Heron and Smyth 2010). Several studies found early evidence of the effectiveness of real-time interventions for a variety of health behaviors, including alcohol use (Cohn et al. 2011; Free et al. 2013; Heron and Smyth 2010; Whittaker et al. 2012). The effectiveness of real-time interventions also seems to be enhanced when EMI is used to deliver tailored content.

### Examples From the Alcohol Literature

The literature on use of EMI to modify alcohol use is relatively small, but growing. As in other areas, such as tobacco use (Abroms et al. 2011) and weight loss (Pagoto et al. 2013), few EMI programs currently available as smartphone apps adhere to evidence-based strategies for treating alcohol dependence (Cohn et al. 2011). However, despite the paucity of studies to date, the real-time delivery of interventions aimed at reducing alcohol consumption holds significant promise to help people who are working to change their drinking behavior (Free et al. 2010, 2013).

Weitzel and colleagues (2007) delivered tailored text messages as EMI in a study of 40 college students who reported drinking alcohol at least once per week. In this well-designed study, both the control and intervention groups used EMA to monitor drinking, allowing the investigators to control for any potential effect of the EMA itself on drinking behavior. Participants in the intervention group also received text messages throughout the day that were tailored to their reported drinking behavior as well as to self-efficacy and outcome expectancies as determined by self-report at baseline. The authors detected small but positive effects of EMI. Specifically, participants in the EMI group reported drinking less, on average, over the study period as well as being more confident in their ability to avoid negative consequences of drinking at the end of the study.

Another example of an EMI used to treat substance use disorders is the A-CHESS system (Gustafson et al. 2014), which is based upon self-determination theory (Ryan and Deci 2000) and Marlatt’s relapse-prevention model (Marlatt 1980). A-CHESS has several functional capabilities, but most relevant to EMI is its ability to deliver tailored text messages based on person-level factors as well as time-varying factors (e.g., level of intrinsic motivation) that are assessed via EMA. A-CHESS also offers a “high-risk-patient locator” that uses EMA to keep track of places where users report they usually obtain alcohol. When the user is near those places, A-CHESS proactively sends a message to raise the user’s awareness of the potential high-risk nature of the location. Users even can configure A-CHESS to alert friends or family when the user is near a high-risk location, thus “crowdsourcing” EMI by alerting individuals who are supporting the person in recovery that the patient may need help, allowing them to reach out to the individual and provide real-time social support. (For more information on A-CHESS and similar EMI-based interventions and their effectiveness, see the article in this issue by Quanbeck and colleagues.)

Although continued research on this kind of preemptive EMI is needed, there is great potential for these interventions to positively impact recovery from AUDs and other behavioral health disorders that are known to be chronic and relapsing in nature. Overall, there seem to be significant opportunities for studying EMIs in the context of AUDs.

## Using EMA and EMI Together

As mentioned at the beginning of this article, the challenges of working with patients with AUDs in outpatient settings are twofold. First, if the provider only can rely on the individual’s self-report of their drinking behavior, the information they obtain likely is biased, inaccurate, and incomplete. Using EMA alone would equip the provider with better information based on which interventions can be offered that are most likely to help the individual stop drinking. However, in this situation, it would still be up to the patient to remember and actively implement the provider’s intervention once they left the clinical encounter and experienced all of the internal and external factors associated with their drinking in their day-to-day life. In other words, there still would be no intervention at the point of choice, resulting in an extremely high risk of failure.

Pairing EMA with EMI can help overcome these challenges. Thus, EMA can be used to monitor the occurrence and patterns of high-risk situations. Based on these data, the timing of the EMI can then be tailored so that real-time intervention occurs at times predicted by EMA data to coincide with high-risk events. EMA can also be used in conjunction with EMI to capture time-varying information that may help tailor the content of the EMI (Riley et al. 2011). For example, based on EMA data that indicate where the individual is when they report an urge to drink (e.g., at home versus at a restaurant) the content of the EMI message sent could be tailored to be most pertinent (e.g., “Consider calling a friend” vs. “Move to a restaurant where alcohol is not served”). Similarly, when EMA data reveal changes in contextual factors (e.g., an individual’s self-efficacy decreases from one day to the next), the content of the EMI can be adjusted to target this contextual factor (e.g., “You will have ups and downs on this journey—take it one day at a time and stay strong!” vs. “Keep up the great work!”) (Weizel et al. 2007). As described in the literature (Chih et al. 2014; Free et al. 2009; Gustafson et al. 2011, 2014; Lagoa et al. 2014; Wetter et al. 2011), EMA protocols can inform EMI schedules in several ways (see the table). Whereas EMI delivered on demand or randomly does not require use of EMA, more sophisticated EMI programs can effectively benefit from data provided via EMA.

It is important to note, however, that as the degree increases to which an EMI delivery schedule relies on EMA, the EMA protocol may become more burdensome for the user, which can reduce compliance. EMI delivery schedules that operate based on predictions made using EMA data likely require a relatively large volume of data on a variety of behavioral and contextual factors to support accurate predictions of high-risk times. Therefore, it is important to balance precision of timing of EMI delivery against the burden imposed on the user. For some individuals, it may be possible to target the EMI to certain times of day when the target behavior is known to occur most likely. In this case, EMA could be used in a more limited capacity for only a short time before, during, and after the high-risk interval, with EMI occurring in response to EMA-detected cues such as location or environmental context (e.g., reports of being around others who are drinking). More often, however, the timing of the target behavior varies and EMA is required to determine when EMI is most likely to be needed. However, EMA and EMI do not have to be conducted simultaneously but may also be done in an asynchronous manner. This means that EMA is used during a baseline observational period to understand the participant’s patterns in the target behavior, and then an EMI schedule is crafted accordingly for subsequent implementation.

Two examples of context-aware EMI studies that use EMA to directly inform the content and timing of EMI delivery are the A-CHESS system discussed above (Gustafson et al. 2011, 2014) and the Mobylize! system (Burns et al. 2011). Mobylize! is a context-aware intervention system for depression that records a combination of EMA-reported mood and context variables with sensors embedded in a smartphone (e.g., light detection, phone usage) to determine when users are in need of EMI. In response to EMA-based reports of low mood in combination with the contextual data sensed by the device, Mobylize! delivers EMI aimed at improving mood or supporting coping.

Perhaps most sophisticated, although as yet least developed, is the use of EMA to inform algorithms that can determine what intervention is needed, and when, and which can continually update over time. In this case, the algorithms use input from EMA data (e.g., when drinking or urges to drink occur, mood, location) to predict when the next target event (e.g., an urge to drink) likely will occur. The algorithm then triggers EMI delivery at the predicted time. The user can confirm whether there actually is a need for the EMI when it is delivered (i.e., whether he or she is in a high-risk situation). Based on this feedback, the “when-to-intervene-with-what” algorithm is continuously updated so that it maintains a high degree of accuracy as the user’s dynamic needs change over time. This approach is used with the Mobylize! System, which involves algorithms to combine user-reported EMA data with information passively sensed by the mobile device to predict when users are experiencing depressed mood (Burns et al. 2011). In the context of alcohol use, EMA could track episodes of drinking, urges to drink, mood, location, and other user-reported or passively sensed (e.g., time of day) data. All this information could continuously be entered into an algorithm that aims to determine the level of urge to drink, which would then trigger intervention delivery at times when urge is predicted to be high. In addition to subsequently asking the user to confirm whether help actually was needed in order to update the algorithm regarding when to deliver EMI, an additional follow-up EMA on urge to drink at some time close to EMI delivery could be used to estimate the effectiveness of the EMI. This information could potentially inform the algorithm on the most effective type of EMI to deliver. These continuous updates based on time-varying data would enable the algorithms to tailor the content and delivery timing of the EMI response to the user’s dynamic experiences of alcohol use and craving over time. Two recent studies (Chih et al. 2014; Lagoa et al. 2014) have addressed ways of predicting user needs in real time, and it is likely that real-time interventions for substance use disorders will increasingly use these techniques.

Despite these potential benefits, the synergistic use of EMA and EMI is challenging for at least two reasons. First, deriving algorithms that can run locally on a mobile device is complicated. Second, even when algorithms can be derived and implemented, they may not always be accurate. This limitation may at least in part be a function of the degree to which the user faithfully participates in EMA—in other words, the algorithms can only be as accurate as the data they receive. Even with highly accurate and complete EMA data, however, there will inevitably be times when algorithm-driven EMI delivery is inaccurate. In this instance, the best-case scenario is that the user receives an EMI when they did not need help, which can either simply be dismissed by the user or may possibly serve to momentarily enhance motivation. At worst, the EMI can act as a cue for the target behavior rather than as a deterrent by prompting the user to think about the behavior when they originally were not. This unintended negative consequence, which also exists with EMI programs that deliver the intervention on a random schedule, needs to be carefully considered.

## Issues Worth Noting

### Challenges and Caveats

EMA and EMI both are exciting and innovative methods that can significantly extend the reach of health care providers working to help individuals overcome addiction and which offer hope of improved success rates for achieving sustained abstinence for people who are attempting to change. By nature, EMA and EMI involve the close observation of an individual’s behavior and, in some cases, attempts to predict specific behaviors. For EMA specifically, it is important to balance the need for gathering a meaningful amount of information against the burden on the respondent to report it. Although compliance with EMA protocols in research studies often is high, even in studies of substance use disorders (Shiffman 2009), this is sometimes accomplished by incentivizing compliance (e.g., Todd et al. 2009). Such incentives are not possible, however, when designing interventions that use EMA in a large population, and therefore special attention should be paid to developing minimally burdensome EMA protocols to avoid declines in compliance. As wearable sensor technology becomes more sophisticated and less intrusive, passively sensed biometric data will perhaps decrease the need to rely on user reports of behavior or affective states (Plarre et al. 2011).

Another concern regarding EMA and EMI are privacy and ethical considerations regarding the collected data (Arora et al., in this issue). The type and level of information gathered from the individual and the inferences that can be drawn about their activities in studies using EMA and/or EMI warrant careful consideration of these issues. This is an ongoing area of discussion in the field of mobile health (Kumar et al. 2013). However, it easily is possible to completely remove identifiers from data collected via EMA and intervention delivered via EMI and to protect the mobile device with a password, thereby greatly reducing risks of privacy violations or breaches of confidentiality. Individuals who begin using EMA and/or are newly exposed to EMI often require training in the procedures (Shiffman 2009). In addition to explaining to participants what they need to do to adhere to EMA protocols and what they can expect regarding EMI, this training offers an opportunity to explain data transmission procedures (e.g., how often EMA data will be procured and by what means) and address any privacy concerns.

Another challenge that also is relevant to the issue of training is designing mobile interfaces that are user friendly and human centered. Even relatively simple EMA protocols or EMI programs will be more engaging and better received by users (and thus, potentially will have greater impact) when they are designed with the user’s needs in mind. Several methods exist for incorporating human-centered aspects into the design of EMA and EMI (e.g., LUMA Institute 2012), and such methods should be considered from the earliest stages of the process of protocol or program design.

Finally, as mentioned previously, reactivity to EMA also needs to be considered. The process of monitoring alone can affect the target behavior, even to a degree that makes it difficult to detect an effect of a separate intervention.

### Analytical Considerations

The data yield from EMA requires careful management and analysis using methods that may not be commonly employed by researchers or practitioners who typically deal with much smaller numbers of data points per patient over a comparable period of time. Although EMA data yield multiple observations per person, the number of observations and their timing also vary across a study population. The repeated observations within an individual also violate some assumptions within traditional regression analyses about independence of observations (Tabachnick 2001). However, many of the same methods that are used in traditional longitudinal research approaches also apply to the analysis of EMA data (Shiffman et al. 2008). EMA data also can be leveraged to great effect using analytic methods from fields of study outside of behavioral medicine, such as engineering (Timms et al. 2014).

When approaching analyses of EMA data, time can be a useful organizing element, because EMA intensively collects data from individuals over extended periods of time. There are multiple ways to consider time in analyses of EMA data, including (but not limited to) calendar time (e.g., time of day, day of week); time elapsed (e.g., time elapsed until first drink); time as a moderator (i.e., examining whether the relationship between two variables changes over time); or excluding time entirely (e.g., analyzing between-subjects differences on a variable of interest that is not time-dependent). Shiffman (2014) has extensively reviewed analytical approaches to EMA data.

## Conclusions

As mobile devices, wearable sensors, and technology-mediated communication have become more ubiquitous in health care, there is increasing evidence that patient-generated data offer enormous potential to improve management of health conditions and health outcomes (Deering 2013). By gathering data and providing intervention in real time, when patients need help most, health care providers are empowered to deliver effective “care between the care” (Backonja et al. 2012). EMA and EMI both offer rigorous approaches that can significantly extend the reach and impact of behavioral-medicine interventions, both of which are greatly needed to make progress in managing hard-to-treat conditions such as AUDs and the behavioral factors that underlie our most pressing public health problems.

## Figures and Tables

**Table:** EMI Schedules and Associated EMA Requirements

EMI Schedule	Required EMA Protocol	Strengths	Limitations	Example
On-demand delivery of real-time intervention	None	Straightforward and simple programming	Relies entirely on the user to ask for help	Gustafson et al. 2011 (includes other schedules as well)
Random delivery of real-time intervention	None	Still relatively simple programming, but capitalizes on potential for intervention delivery to happen at moments of high risk	Intervention may occur at times when help is not required and may not occur at high-risk times; potential unintended negative consequence of prompting the behavior	Free et al. 2009
Delivery in response to user-reported contextual data (e.g., urge, mood)	Assessment of contextual factors on a regular basis, using both system-initiated and user-initiated reports	Enables delivery of real-time intervention in response to contextual factors associated with increased probability of high-risk events	EMA protocol adherence creates respondent burden; system-initiated prompts may not coincide with high-risk events	Wetter et al. 2011
Delivery in response to passively sensed contextual data (e.g., location)	Continuous sensing of passively collected contextual data	Minimal participant burden; intervention is delivered “seamlessly”	Relationship between passively sensed data and probability of self-regulatory failure must be accurate; potential unintended negative consequence of prompting the behavior; continuous sensing places high demand on battery and data capabilities of the mobile device	Gustafson et al. 2014
Delivery in response to model predictions of high-risk times based on user-reported and passively sensed data	Assessment of contextual factors on a regular basis, using both system-initiated and user-initiated reports and continuous sensing of passively collected contextual data	Enables delivery of real-time intervention in response to contextual factors associated with increased probability of self-regulatory failure; reduced participant burden; intervention is delivered “seamlessly”	Requires algorithm development and implementation as well as burden on capabilities of the mobile device (data, battery) and user (EMA protocol adherence)	Chih et al. 2014 (development); Lagoa et al. 2014 (development)

## References

[b1-arcr-36-1-9] Abroms LC, Padmanabhan N, Thaweethai L, Phillips T (2011). iPhone apps for smoking cessation: A content analysis. American Journal of Preventive Medicine.

[b2-arcr-36-1-9] American Psychiatric Association (2013). Diagnostic and Statistical Manual of Mental Disorders.

[b3-arcr-36-1-9] Backonja U, Kim K, Casper GR (2012). Observations of daily living: Putting the “personal” in personal health records. Nursing Informatics.

[b4-arcr-36-1-9] Ball SA, Todd M, Tennen H (2007). Brief motivational enhancement and coping skills interventions for heavy drinking. Addictive Behaviors.

[b5-arcr-36-1-9] Bradburn NM, Rips LJ, Shevell SK (1987). Answering autobiographical questions: The impact of memory and inference on surveys. Science.

[b6-arcr-36-1-9] Brandon TH, Vidrine JI, Litvin EB (2007). Relapse and relapse prevention. Annual Reviews in Clinical Psychology.

[b7-arcr-36-1-9] Burns MN, Begale M, Duffecy J (2011). Harnessing context sensing to develop a mobile intervention for depression. Journal of Medical Internet Research.

[b8-arcr-36-1-9] Chih MY, Patton T, McTavish FM (2014). Predictive modeling of addiction lapses in a mobile health application. Journal of Substance Abuse Treatment.

[b9-arcr-36-1-9] Cohn AM, Hunter-Reel D, Hagman BT, Mitchell J (2011). Promoting behavior change from alcohol use through mobile technology: The future of ecological momentary assessment. Alcoholism: Clinical and Experimental Research.

[b10-arcr-36-1-9] Collins RL, Morsheimer ET, Shiffman S (1998). Ecological momentary assessment in a behavioral drinking moderation training program. Experimental and Clinical Psychopharmacology.

[b11-arcr-36-1-9] Deering MJ (2013). Issue Brief: Patient-Generated Health Data and Health IT.

[b12-arcr-36-1-9] Ferguson SG, Shiffman S (2011). Using the methods of ecological momentary assessment in substance dependence research—Smoking cessation as a case study. Substance Use & Misuse.

[b13-arcr-36-1-9] Free C, Phillips G, Felix L (2010). The effectiveness of M-health technologies for improving health and health services: A systematic review protocol. BMC Research Notes.

[b14-arcr-36-1-9] Free C, Phillips G, Galli L (2013). The effectiveness of mobile-health technology-based health behaviour change or disease management interventions for health care consumers: A systematic review. PLoS Medicine.

[b15-arcr-36-1-9] Free C, Whittaker R, Knight R (2009). Txt2stop: A pilot randomised controlled trial of mobile phone-based smoking cessation support. Tobacco Control.

[b16-arcr-36-1-9] Gurvich EM, Kenna GA, Leggio L (2013). Use of novel technology-based techniques to improve alcohol-related outcomes in clinical trials. Alcohol and Alcoholism.

[b17-arcr-36-1-9] Gustafson DH, McTavish FM, Chih MY (2014). A smartphone application to support recovery from alcoholism: A randomized clinical trial. JAMA Psychiatry.

[b18-arcr-36-1-9] Gustafson DH, Shaw BR, Isham A (2011). Explicating an evidence-based, theoretically informed, mobile technology-based system to improve outcomes for people in recovery for alcohol dependence. Substance Use & Misuse.

[b19-arcr-36-1-9] Hawkins RP, Kreuter M, Resnicow K (2008). Understanding tailoring in communicating about health. Health Education Research.

[b20-arcr-36-1-9] Heron KE, Smyth JM (2010). Ecological momentary interventions: Incorporating mobile technology into psychosocial and health behaviour treatments. British Journal of Health Psychology.

[b21-arcr-36-1-9] Holt LJ, Litt MD, Cooney NL (2012). Prospective analysis of early lapse to drinking and smoking among individuals in concurrent alcohol and tobacco treatment. Psychology of Addictive Behaviors.

[b22-arcr-36-1-9] Hufford MR, Shields AL, Shiffman S (2002). Reactivity to ecological momentary assessment: An example using undergraduate problem drinkers. Psychology of Addictive Behaviors.

[b23-arcr-36-1-9] International Data Corporation (2013). Always Connected: How Smartphones and Social Keep Us Engaged.

[b24-arcr-36-1-9] Kashdan TB, Ferssizidis P, Collins RL, Muraven M (2010). Emotion differentiation as resilience against excessive alcohol use: An ecological momentary assessment in underage social drinkers. Psychological Science.

[b25-arcr-36-1-9] Kumar S, Nilsen WJ, Abernethy A (2013). Mobile health technology evaluation: The mHealth evidence workshop. American Journal of Preventive Medicine.

[b26-arcr-36-1-9] Lagoa CM, Bekiroglu K, Lanza ST, Murphy SA (2014). Designing adaptive intensive interventions using methods from engineering. Journal of Consulting and Clinical Psychology.

[b27-arcr-36-1-9] Loewenstein G (2005). Hot-cold empathy gaps and medical decision making. Health Psychology.

[b28-arcr-36-1-9] LUMA Institute (2012). Innovating for People: Handbook of Human-Centered Design Methods.

[b29-arcr-36-1-9] Marlatt GA, Gordon JR, Davidson SM, Davidson PO (1980). Determinants of relapse: Implications for the maintenance of behavior change. Behavioral Medicine: Changing Health Lifestyles.

[b30-arcr-36-1-9] Miller WR (1996). What is a relapse? Fifty ways to leave the wagon. Addiction.

[b31-arcr-36-1-9] Muraven M, Collins RL, Morsheimer ET (2005). One too many: Predicting future alcohol consumption following heavy drinking. Experimental and Clinical Psychopharmacology.

[b32-arcr-36-1-9] Noar SM, Benac CN, Harris MS (2007). Does tailoring matter? Meta-analytic review of tailored print health behavior change interventions. Psychological Bulletin.

[b33-arcr-36-1-9] Pagoto S, Schneider K, Jojic M (2013). Evidence-based strategies in weight-loss mobile apps. American Journal of Preventive Medicine.

[b34-arcr-36-1-9] Patrick K, Griswold WG, Raab F, Intille SS (2008). Health and the mobile phone. American Journal of Preventive Medicine.

[b35-arcr-36-1-9] Piasecki TM, Wood PK, Shiffman S (2012). Responses to alcohol and cigarette use during ecologically assessed drinking episodes. Psychopharmacology (Berlin).

[b36-arcr-36-1-9] Plarre K, Raij A, Hossain SM Continuous Inference of Psychological Stress From Sensory Measurements Collected in the Natural Environment.

[b37-arcr-36-1-9] Ramirez J, Miranda R (2014). Alcohol craving in adolescents: Bridging the laboratory and natural environment. Psychopharmacology (Berlin).

[b38-arcr-36-1-9] Reis HT, Gosling SD, Gilbert DT, Fiske ST, Lindzey G (2010). Social psychological methods outside the laboratory. Handbook of Social Psychology.

[b39-arcr-36-1-9] Riley WT, Rivera DE, Atienza AA (2011). Health behavior models in the age of mobile interventions: Are our theories up to the task?. Translational Behavioral Medicine.

[b40-arcr-36-1-9] Ryan RM, Deci EL (2000). Self-determination theory and the facilitation of intrinsic motivation, social development, and well-being. American Psychologist.

[b41-arcr-36-1-9] Serre F, Fatseas M, Debrabant R (2012). Ecological momentary assessment in alcohol, tobacco, cannabis and opiate dependence: A comparison of feasibility and validity. Drug and Alcohol Dependence.

[b42-arcr-36-1-9] Shiffman S (2009). Ecological momentary assessment (EMA) in studies of substance use. Psychological Assessment.

[b43-arcr-36-1-9] Shiffman S (2014). Conceptualizing analyses of ecological momentary assessment data. Nicotine & Tobacco Research.

[b44-arcr-36-1-9] Shiffman S, Paty JA, Gnys M (1996). First lapses to smoking: Within-subjects analysis of real-time reports. Journal of Consulting and Clinical Psychology.

[b45-arcr-36-1-9] Shiffman S, Stone AA, Hufford MR (2008). Ecological momentary assessment. Annual Review of Clinical Psychology.

[b46-arcr-36-1-9] Smyth JM, Stone AA (2003). Ecological momentary assessment research in behavioral medicine. Journal of Happiness Studies.

[b47-arcr-36-1-9] Stone AA, Shiffman S (1994). Ecological momentary assessment (EMA) in behavioral medicine. Annals of Behavioral Medicine.

[b48-arcr-36-1-9] Stone AA, Shiffman S (2002). Capturing momentary, self-report data: A proposal for reporting guidelines. Annals of Behavioral Medicine.

[b49-arcr-36-1-9] Stone AA, Shiffman S, Atienza AA, Nebeling L, Stone AA, Shiffman S, Atienza AA, Nebeling L (2007). Historical roots and rationale of ecological momentary assessment (EMA). The Science of Real-Time Data Capture: Self-Reports in Health Research.

[b50-arcr-36-1-9] Strecher VJ, Shiffman S, West R (2005). Randomized controlled trial of a web-based computer-tailored smoking cessation program as a supplement to nicotine patch therapy. Addiction.

[b51-arcr-36-1-9] Tabachnick BG, Fidell LS (2001). Using Multivariate Statistics.

[b52-arcr-36-1-9] Timms KP, Rivera DE, Collins LM, Piper ME (2014). A dynamical systems approach to understand self-regulation in smoking cessation behavior change. Nicotine & Tobacco Research.

[b53-arcr-36-1-9] Todd M, Armeli S, Tennen H (2009). Interpersonal problems and negative mood as predictors of within-day time to drinking. Psychology of Addictive Behaviors.

[b54-arcr-36-1-9] Voogt CV, Kuntsche E, Kleinjan M (2013). Using ecological momentary assessment in testing the effectiveness of an alcohol intervention: A two-arm parallel group randomized controlled trial. PLoS One.

[b55-arcr-36-1-9] Weitzel JA, Bernhardt JM, Usdan S (2007). Using wireless handheld computers and tailored text messaging to reduce negative consequences of drinking alcohol. Journal of Studies on Alcohol and Drugs.

[b56-arcr-36-1-9] Wetter DW, McClure JB, Cofta-Woerpel L (2011). A randomized clinical trial of a palmtop computer-delivered treatment for smoking relapse prevention among women. Psychology of Addictive Behaviors.

[b57-arcr-36-1-9] Whittaker R, Borland R, Bullen C (2009). Mobile phone-based interventions for smoking cessation. Cochrane Database of Systematic Reviews.

[b58-arcr-36-1-9] Whittaker R, McRobbie H, Bullen C (2012). Mobile phone-based interventions for smoking cessation. Cochrane Database of Systematic Reviews.

[b59-arcr-36-1-9] Witkiewitz K, Marlatt GA (2004). Relapse prevention for alcohol and drug problems: That was Zen, this is Tao. American Psychologist.

